# MicroRNA Expression Profile in Penile Cancer Revealed by Next-Generation Small RNA Sequencing

**DOI:** 10.1371/journal.pone.0131336

**Published:** 2015-07-09

**Authors:** Li Zhang, Pengfei Wei, Xudong Shen, Yuanwei Zhang, Bo Xu, Jun Zhou, Song Fan, Zongyao Hao, Haoqiang Shi, Xiansheng Zhang, Rui Kong, Lingfan Xu, Jingjing Gao, Duohong Zou, Chaozhao Liang

**Affiliations:** 1 Department of Urology, The First Affiliated Hospital of Anhui Medical University, Hefei, Anhui, China; 2 Institute of Urology, Anhui Medical University, Hefei, Anhui, China; 3 School of Life Sciences, University of Science and Technology of China, Hefei, Anhui, China; 4 Center for Reproductive Medicine, Anhui Provincial Hospital Affiliated to Anhui Medical University, Hefei, Anhui, China; 5 Key Laboratory of Oral Diseases Research of Anhui Province, Stomatologic College, Anhui Medical University, Hefei, Anhui, China; Huazhong University of Science and Technology, CHINA

## Abstract

Penile cancer (PeCa) is a relatively rare tumor entity but possesses higher morbidity and mortality rates especially in developing countries. To date, the concrete pathogenic signaling pathways and core machineries involved in tumorigenesis and progression of PeCa remain to be elucidated. Several studies suggested miRNAs, which modulate gene expression at posttranscriptional level, were frequently mis-regulated and aberrantly expressed in human cancers. However, the miRNA profile in human PeCa has not been reported before. In this present study, the miRNA profile was obtained from 10 fresh penile cancerous tissues and matched adjacent non-cancerous tissues via next-generation sequencing. As a result, a total of 751 and 806 annotated miRNAs were identified in normal and cancerous penile tissues, respectively. Among which, 56 miRNAs with significantly different expression levels between paired tissues were identified. Subsequently, several annotated miRNAs were selected randomly and validated using quantitative real-time PCR. Compared with the previous publications regarding to the altered miRNAs expression in various cancers and especially genitourinary (prostate, bladder, kidney, testis) cancers, the most majority of deregulated miRNAs showed the similar expression pattern in penile cancer. Moreover, the bioinformatics analyses suggested that the putative target genes of differentially expressed miRNAs between cancerous and matched normal penile tissues were tightly associated with cell junction, proliferation, growth as well as genomic instability and so on, by modulating Wnt, MAPK, p53, PI3K-Akt, Notch and TGF-β signaling pathways, which were all well-established to participate in cancer initiation and progression. Our work presents a global view of the differentially expressed miRNAs and potentially regulatory networks of their target genes for clarifying the pathogenic transformation of normal penis to PeCa, which research resource also provides new insights into future investigations aimed to explore the in-depth mechanisms of miRNAs and other small RNAs including piRNAs in penile carcinogenesis regulation and effective target-specific theragnosis.

## Introduction

Molecular research has emerged as a promising tool for in-depth understanding into carcinogenesis, with its cellular components, signaling pathways and pharmaceutical targets in cancer therapeutics. Unfortunately, the remarkable progress has not encompassed all cancer types. Penile cancer (PeCa) is such a relatively rare tumor entity within the developed countries of the world, only about 1,640 penile cancer cases were newly diagnosed and 320 cancer-related deaths were estimated in 2014 in the United States nationally [1]. Whereas, the morbidity and mortality rates of PeCa in most developing countries were considerably higher, accounting for a 3–10 fold increase at the rates of developed countries over the past decades [2]. Penile cancer is associated with several established risk factors such as human papillomavirus (HPV) infection, poor hygiene, phimosis with chronic inflammation, smoking and some epigenetic alternations including histone methylation modifications [3,4]. Besides, a few genes during carcinogenesis, proliferation, invasion and metastasis of PeCa have been identified. For instance, p53, cyclin-dependent kinase inhibitor 2A (CDKN2A) and matrix metallopeptidase 9 (MMP-9) were proven to play crucial roles in PeCa carcinogenic routes and epithelial-mesenchymal transition (EMT), respectively [5]. Nonetheless, as mentioned above, additional comprehensive research to further elucidate the exact mechanisms of molecular changes underlying the initiation, development and progression of PeCa is imperative for us to explore novel and valuable preventive, early detection, targeted therapies and prognosis prediction approaches for this genitourinary disorder.

Mature microRNAs (miRNAs) are a group of endogenously expressed, evolutionally conserved, single-stranded and non-coding small RNAs (smRNAs, approximately 19–23 nucleotides in length) that act as posttranscriptional regulators of gene expression in a wide range of organisms [6]. To date, more than 1000 human miRNAs have been identified and tremendous observations have been achieved in connecting the alternative and aberrant expression levels of miRNAs with the initiation and progression of human diseases, especially for various kinds of cancer types [7–9], as no less than half miRNA genes are located in cancer-associated regions or fragile sites in the whole genome [10]. miRNA deregulation in tumor biology was first observed in miRNA-15a and miRNA-16-1 within locus 13q14, both of the two miRNAs which targeted B-cell lymphoma 2 (Bcl-2) mRNA were deleted or downregulated in most majority of the chronic lymphocytic leukemia (CLL) cases, resulting in tumorigenesis by reducing apoptotic activities [11]. Presently, it has been widely accepted that distinct cancer types, stages, regression grades or differentiation states might possess individual miRNA expression profiles, which hold great promise in acting as reliable molecular biomarkers for cancer diagnosis and may also reveal new pathogenic signaling pathways correlated with carcinogenesis and progression for targeted molecular therapeutics [12]. Ever since, a variety of techniques have been introduced to conduct miRNA profiling. For instance, quantitative reverse-transcription polymerase chain reaction (qRT-PCR) assays, Northern blotting analyses and microarrays have ever been extensively used for miRNA profiling studies [13]. Recently, a newly developed technology named next-generation sequencing (NGS) has attracted much attention in small RNA (smRNA) profiling due to its unique advantages in test specificity, sensitivity and novel smRNAs identification [14–16]. Through which, we can detect almost all smRNAs such as known and novel miRNAs even with extremely low abundance, small cytoplasmic RNAs (scRNAs), nuclear RNAs (snRNAs), nucleolar RNAs (snoRNAs) and piwi-interacting RNAs (piRNAs) [17].

In the present study, we aimed at filling the gap in information regarding the miRNA profile in human penile cancer and evaluated a possible correlation between differentiated miRNA expression levels with tumorigenesis and progression. By applying next-generation sequencing (NGS) technology, we performed smRNA-seq for ten paired fresh-frozen specimens of penile squamous cell carcinoma and matched histologically normal penile tissues which were adjacent to the cancer. We have addressed the general information of the miRNA expression profiles in both the paired penile tissues and especially found that a number of miRNAs were aberrantly expressed in penile cancers compared with matched normal tissues. Notably, the gene ontology (GO) analysis of potential miRNA target genes indicated that deregulated miRNAs in enriched biological processes, molecular functions and cellular components were involved in cell growth, cell shape, axonogenesis, protein activity regulation and angiogenesis, which together participated in the transformation of normal cells to malignant lesions. Besides, the kyoto encyclopedia of genes and genomes (KEGG) pathway analysis successfully enriched several tightly cancer-associated pathways which were all well-documented to participate in cancer initiation, development, invasion, metastasis and also promising therapy. Our results provide a valuable research resource to further elucidate the regulatory roles of miRNAs in penile tumorigenesis and progression, which also holds great promise in facilitating the development of novel diagnostic biomarkers and effective therapeutic strategies for PeCa.

## Materials and Methods

### Ethics statement

Informed consents were signed by all the thirteen patients themselves with penile squamous cell carcinoma who had received partial penectomy and the investigation was evaluated thoroughly and then approved strictly according to the regulations by the Ethics Committees on Human Research of the First Affiliated Hospital of Anhui Medical University (Approval No. PJ20140906).

### Clinical specimens collection

Penile cancer and matched non-malignant specimens were collected from a total of 13 patients who underwent partial penectomy from 2013 to 2015 at Department of Urology, the First Affiliated Hospital of Anhui Medical University. Immediately after surgery, cancerous and matched normal penile tissues were separately stored in RNAlater solution (Ambion, USA) and frozen at -80°C according to a standard operation procedure (SOP) guided by the uropathologist. Briefly, tumor tissues containing more than 80% tumorous lesions were included for further analyses. Correspondingly, the matched normal adjacent tissues were obtained from where at least 1.0 cm apart from the visible tumorous tissues in penis. Each cancerous and non-malignant tissues were diagnosed histopathologically by Hematoxylin-eosin (HE)-staining.

### SmRNA library construction and deep sequencing

Total RNA was collected from ten pairs of fresh-frozen penile cancers and matched histologically normal penile tissues by using TRIzol reagent (Life Technologies, USA). Qualified RNA with A260/A280 ratio more than 1.8 was subsequently assessed for RNA integrity by conducting the DNA chip assay. Two smRNA libraries were constructed from pooled and integrated RNAs of ten individuals for each group (cancerous and matched normal penile tissues) by using a smRNA sample prep kit (Illumina, USA) following the standard protocols with specific modifications [15]. Briefly, the appropriate fractions ranged from 18 nt to 28 nt were separated, purified via 15% (w/v) denaturing polyacrylamide gel electrophoresis and then linked to RNA adaptors (5’-GTTCAGAGTTCTACAGTCCGACGATC and 3’-TCGTATGCCGTCTTCTGCTTG) followed by RT-PCR amplification. Next, the PCR products were further purified on agarose gels to establish the libraries. Finally, the two libraries which constructed from ten cancerous penile tissues (pooled and homogenized to a library) and ten matched adjacent normal penile tissues (pooled and homogenized to another library) were sequenced by applying Illumina Hiseq 2000 (Illumina, USA) at Beijing Genomics Institute (Shenzhen, China) strictly following the standard protocols [18].

### NGS data analyses and novel miRNAs identification

The smRNA sequencing data were further analyzed referring to the methods described in the previous publications [15–18]. Concretely, after deleting 5’ adaptor and trimming 3’ acceptor sequences, removing contaminated and low-quality reads (Q<10, the quality value was calculated by Q = ASCII character code-64) such as reads without insert fragment or containing poly(A) stretches, the qualified unique tags were mapped to GRCh37.p5 by introducing SOAP2.0 ultrafast tool (http://soap.genomics.org.cn) [19]. Tags with no more than one mismatch were picked out for subsequent analyses. The conserved known miRNAs were identified by aligning the mapped tags to miRBase tool (version 18.0) in *Homo sapiens* [20], and the tags not matched in miRBase were further aligned against the sequences of other non-coding RNAs (rRNAs, tRNAs, snRNAs, snoRNAs) presented on Rfam database (http://www.sanger.ac.uk/resources/databases/rfam.html) [21], repeats database as well as piRNA database [22]. In consideration of a few smRNA tags might map to more than one category, we followed the priority rules described in the previous publication to guarantee that every unique smRNA was mapped to unique annotation as follows: miRNA, Rfam, repeats, piRNA and mRNA (122.228.158.106/mr2_dev) [23].

After identifying the known miRNAs, the remaining sequences from all cancerous and matched normal penile specimens not annotated by the above-mentioned databases were picked out of the two libraries to be aligned with the integrated human transcriptome for predicting novel miRNAs. All hairpin-like structures containing unclassified smRNA tags consisting of no less than 45 reads were predicted using classical pre-microRNAs prediction tool Mireap (http://sourceforge.net/projects/mireap/) [24] strictly according to the following rules: 1) Length of miRNA sequences range from 18 to 26 nt; 2) Free energy of a miRNA precursor is less than -18 kcal/mol; 3) Bulge allowed for miRNA and the paired precursor miRNA* is less than 4; 4) Space between miRNA and the paired precursor miRNA* is no more than 35 nt. Meanwhile, RNA Fold (http://rna.tbi.univie.ac.at/RNA/RNAfold.html) [25], a program computes the minimum free energy (MFE) and backtraces an optimal secondary structure, was lent to predict all the essential secondary structures of potential miRNA precursors. All the raw data generated by next generation sequencing has been deposited in the designated database ArrayExpress (Accession number: E-MTAB-3087).

### Bioinformatics analyses for differentially expressed miRNAs

The approaches described in our previous publication for bioinformatics analyses were introduced to perform the target genes prediction in the present study [17–18]. Briefly, the targeted genes of differentially expressed miRNAs from cancerous and matched normal penile tissues were predicted using MicroCosm Targets, formerly called miRBase targets (http://www.ebi.ac.uk/enright-srv/microcosm/) [26–27], TargetSpy (http://targetspy.org/) [28] and miRanda (http://www.microrna.org) [29]. The concrete predictions were strictly according to the rules described as follows: 1) Any mismatch should not be found at the seed region; 2) The free energy of the miRNA/target duplex should be less than -20 Kcal/mol; 3) The total score for a miRNA-mRNA pair should exceed 140 [17–18].

Subsequently, the enriched GO terms (http://www.geneontology.org) [30] and KEGG pathways (http://www.genome.jp/kegg/) [31] analyses were conducted for the predicted target genes of differentially expressed miRNAs from normal and cancerous penile specimens. Concretely, after mapping the potential target genes to the dataset of GO annotations and KEGG pathways, sorting out the enriched biological processes and signaling pathways through Hypergeometric and Fisher's tests, the enrichment ratios and p-values for GO and KEGG were calculated via introducing the identical formula mentioned in our previous report [17], then the False Discovery Rate (FDR) adjustment was performed to judge the significance of differences in multiple testing [32]. Eventually, the key GO terms and pathways were identified only under the circumstance that enrichment ratio≥2.0 and FDR p-value<0.05 simultaneously.

### qRT-PCR verification for altered miRNA expression

Mature miRNAs quantification was performed by routine quantitative real-time PCR using an Applied Biosystems StepOne Real-Time PCR System (Applied Biosystems, USA) and a SYBR premix Ex-Taq II kit (Takara, Japan) with optimized reaction conditions and using the specific primers listed in S1 Table. Briefly, total RNA was extracted from 13 paired cancerous and matched normal penile tissues. Among which, the extracted RNA from the identical ten patients applied to NGS was pooled and validated, while each total RNA from another three patients was separately used for further analyses. After the cDNA synthesis, triplicate reactions were performed at 95°C/10 min, the subsequent 40 amplified cycles were conducted at 95°C/15 s and 60°C/60 s. Meanwhile, U6 snRNA was lent as an internal control to normalize the miRNA expression. For data analysis, the threshold cycle (Ct) was defined as the fractional cycles when the fluorescence passed a fixed threshold and further applied to calculate relative miRNA abundances (△△Ct = Ct_miRNA_-Ctsn_RNAU6_). Relative expression fold changes were calculated using 2^-△△^Ct formula [16]. The miRNA concentration differences between cancerous and normal penile tissues were analyzed using unpaired t-tests [18]. When a p-value was found to be less than 0.05, it would be considered statistically significant.

## Results

### Overview of whole genome smRNA-sequencing data from the paired penile specimens

**Fig 1:**
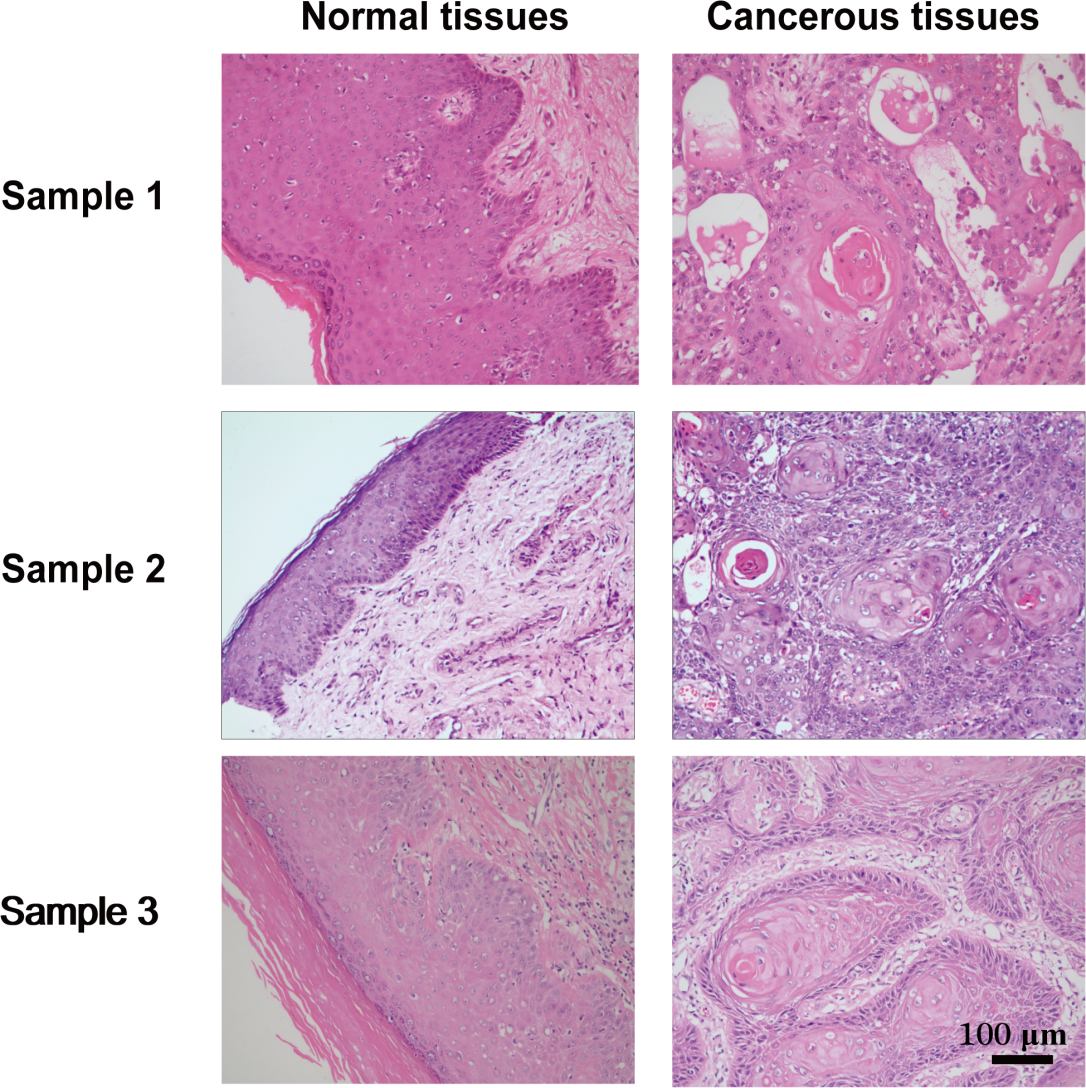
Hematoxylin-eosin (HE) staining of penile tissues from patients who underwent a partial penectomy. HE staining illustrated the representative adjacent normal penile tissues (left) and cancerous penile specimens (right) from three patients, respectively.

All smRNAs of 18–32 nucleotides from the paired fresh-frozen specimens of penile squamous cell carcinoma (hereafter referred to as “cancerous tissues”) and matched histologically normal penile tissues adjacent to the cancer (hereafter referred to as “normal tissues”), which have been diagnosed histopathologically by HE-staining (Fig 1), were deep-sequenced in order to obtain a comprehensive view of smRNAs profile.

**Fig 2:**
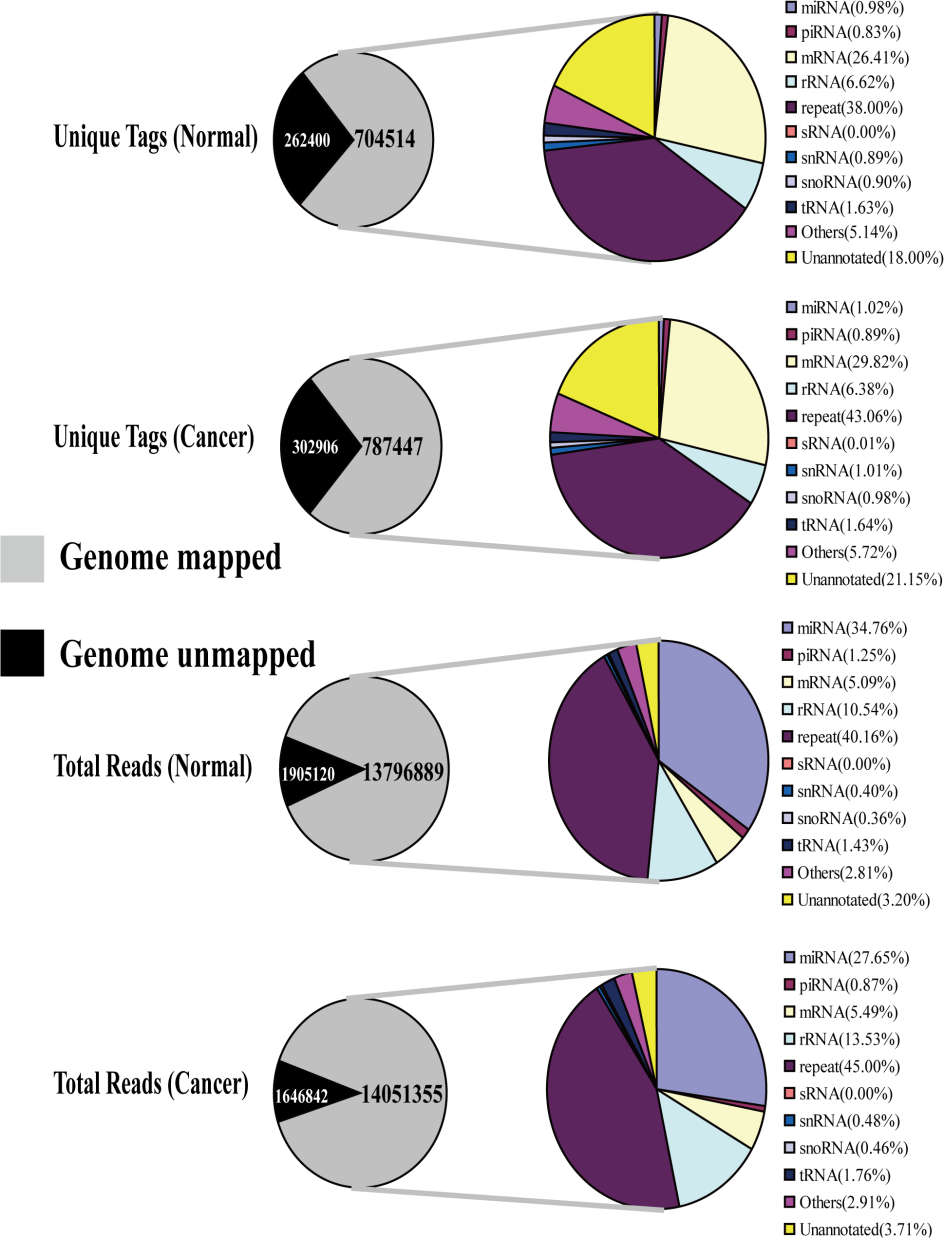
Results of geneome mapping and distribution of RNAs among different categories. The unique tags and total reads aligned to the human genome sequence dataset were obtained. The mapped unique tags and clean reads were annotated and classified as miRNA, piRNA, rRNA, sRNA, snRNA, snoRNA, tRNA, repeats, etc. based on comparison with analytical databases, partial tags and reads were not annotated.

For each sample, 13,796,889 (out of 15,702,009 reads) and 14,051,355 sequence reads (out of 15,698,197 reads) aligned to the human genome sequence dataset were obtained, representing 704,514 (out of 966,914) and 787,447 (out of 1,090,353) unique tags, respectively (S2 Table). Among which, 6,898 unique tags corresponding to 4,795,955 reads and 7,173 unique tags corresponding to 3,815,482 reads were matched to known miRNAs for normal and cancerous tissues, respectively. Meanwhile, 5,877 corresponding to 172,216 reads and 6,272 corresponding to 119,724 reads were matched to known piRNAs for normal and cancerous tissues, respectively. The rest of the sequences were matched into other types of RNAs, including mRNAs, rRNAs, sRNAs, snRNAs, snoRNAs, tRNAs, repeats and so on (Fig 2 and S2 Table). Among which, the repeats mainly consisted of rRNA either based on total reads or unique tags (S3 Table).

The majority of these clean reads were 22 nt in size, consistently followed by 23-nt and 21-nt RNA fragments (S1 Fig). Furthermore, to understand the distribution pattern of all the unique tags on chromosomes, the detailed location of each tag on chromosome was evaluated. As a result, the chromosome distributions between the cancerous and normal tissues were generally the same. Concretely, in the cancerous penile tissues, chromosome 1 harbored most of the unique tags, followed by chromosome 2, 11, 12 and 17, correspondingly, most of the unique tags located on chromosome 1, 2, 17, 12, 11 in the adjacent normal penile tissues (S2 Fig).

### Characteristics of top abundant known and novel miRNAs

To analyze the most abundant miRNAs in penile tissues, we listed the top 20 highly expressed miRNAs with relatively more reads count in decreasing order out of the 751 and 806 known miRNAs in normal and cancerous penile tissues, respectively, which expression profile was indicated by both the absolute reads count and the reads count out of per million reads count. In normal penile tissues, let-7a-5p, let-7f-5p, let-7b-5p, let-7c-5p, miR-140-3p, let-7g-5p, let-7e-5p, miR-103a-3p, miR-320a, miR-143-3p were the top abundant miRNAs. Correspondingly, the highly expressed miRNAs in cancerous penile tissues were let-7f-5p, let-7a-5p, let-7b-5p, let-7c-5p, miR-140-3p, miR-199b-3p, let-7g-5p, miR-199a-3p, let-7e-5p and miR-143-3p possessed the top abundant expression levels. From which, we found that the most highly expressed miRNAs in normal and cancerous penile tissues were approximately the same (Table 1), indicating the identically histological origin of the paired samples undergoing deep sequencing.

**Table 1:** The top 20 most abundant known miRNAs expressed in adjacent normal and cancerous penile tissues.

miRNA name	Adjacent normal penile tissues		miRNA name	Cancerous penile tissues	
	Absolute count	Relative count (rpm^a^)		Absolute count	Relative count (rpm^a^)
hsa-let-7a-5p	1429678	103623.2153	hsa-let-7f-5p	1129415	80377.6575
hsa-let-7f-5p	1173964	85089.0371	hsa-let-7a-5p	983493	69992.7516
hsa-let-7b-5p	939706	68109.9920	hsa-let-7b-5p	548313	39022.0730
hsa-let-7c-5p	305496	22142.3830	hsa-let-7c-5p	274264	19518.6870
hsa-miR-140-3p	83092	6022.5171	hsa-miR-140-3p	89204	6348.4269
hsa-let-7g-5p	63472	4600.4574	hsa-miR-199b-3p	62714	4463.1995
hsa-let-7e-5p	58201	4218.4147	hsa-let-7g-5p	61200	4355.4518
hsa-miR-103a-3p	56867	4121.7263	hsa-miR-199a-3p	57539	4094.9076
hsa-miR-320a	54859	3976.6211	hsa-let-7e-5p	35209	2505.7370
hsa-miR-143-3p	38934	2821.9405	hsa-miR-143-3p	32888	2340.5572
hsa-miR-199a-3p	35860	2599.13667	hsa-let-7i-5p	32786	2333.2981
hsa-miR-199b-3p	34032	2466.6430	hsa-miR-107	31730	2258.1452
hsa-miR-191-5p	30643	2221.0079	hsa-miR-1	30739	2187.6182
hsa-miR-423-5p	28638	2075.6853	hsa-miR-103a-3p	29473	2097.5201
hsa-miR-145-5p	27272	1976.6775	hsa-let-7d-5p	26475	1884.1599
hsa-let-7d-5p	27036	1959.5722	hsa-miR-320a	20787	1479.3591
hsa-miR-1	23534	1705.7469	hsa-miR-191-5p	20608	1466.6201
hsa-miR-205-5p	22994	1666.6076	hsa-miR-101-3p	19697	1401.7865
hsa-miR-23b-3p	21745	1576.0799	hsa-miR-26a-5p	18164	1292.6867
hsa-miR-26a-5p	20912	1515.7040	hsa-miR-26b-5p	17965	1278.5244

^a^rpm: reads count per million reads count

To identify potential novel miRNAs in penile tissues, the unclassified tags were further processed using Mireap tool. Only those tags with reads count more than 50 and strictly matching the default parameters were classified as candidates of novel mature miRNAs. On the basis of this analysis, each 10 miRNA precursors (whose potential stem-loop structures were shown in S3 Fig) encoding the most abundant predicted novel mature miRNA candidates with lengths ranged from 22 to 24 nt were separately identified in the two smRNA libraries from penile tissues. Notably, 7 abundantly expressed novel miRNAs were overlapped between cancerous and matched adjacent normal penile tissues (Table 2), which indicated the identically histological origin and chromosomal location of the miRNAs in both tissues once again. Meanwhile, these novel miRNAs originated from same precursors have also shown heterogeneity and preference, by generating relatively more mature products from the 3’ ends compared with the 5’ ends and among the top 10 novel mature miRNAs in either penile tissues, 9 miRNAs initiated with adenine or uridine (Table 2), which patterns were consistent with the previous report [17].

**Table 2:** The list of top 10 most abundant novel miRNAs expressed in adjacent normal penile tissues and cancerous penile tissues.

Adjacent normal penile tissues			
miRNA ID	Mature Sequence	Reads count	Location of novel miRNA precusor
m0093-3p	AAAAGCUGGGUUGAGAGGGCAAA	26176	chr1:224444740:224444817:-
m0157-3p	AAAAGCUGGGUUGAGAGGGCGAA	8275	chr8:22102477:22102554:-
m0005-3p	AGCAGCAUUGUACAGGGCUAUCA	4928	chr10:91352503:91352582:-
m0115-5p	UGAGGUAGUAGUUUGUACAGUUU	4545	chr3:52302283:52302383:-
m0065-3p	AUCACAUUGCCAGGGAUUUCCAA	4423	chr19:13947397:13947476:-
m0061-5p	CACCCGUAGAACCGACCUUGCG	2951	chr19:52195861:52195939:+
m0011-5p	UCUACAGUGCACGUGUCUCCAGU	1720	chr11:72326100:72326178:-
m0063-3p	UGGCUCAGUUCAGCAGGAACAGGG	1497	chr19:13947091:13947177:-
m0178-3p	AGCUACAUUGUCUGCUGGGUUUC	1153	chrX:45605598:45605680:-
m0179-3p	AGCUACAUCUGGCUACUGGGUCU	928	chrX:45606430:45606513:-
**Cancerous penile tissues**			
**miRNA ID**	**Mature Sequence**	**Reads count**	**Location of novel miRNA precusor**
m0101-3p	AAAAGCUGGGUUGAGAGGGCAAA	9846	chr1:224444740:224444817:-
m0133-5p	UGAGGUAGUAGUUUGUACAGUUU	7509	chr3:52302288:52302383:-
m0005-3p	AGCAGCAUUGUACAGGGCUAUCA	3232	chr10:91352503:91352585:-
m0070-5p	CACCCGUAGAACCGACCUUGCG	2481	chr19:52195861:52195939:+
m0174-3p	AAAAGCUGGGUUGAGAGGGCGAAA	1857	chr8:22102476:22102556:-
m0095-3p	AAAAGCUGGGUUGAGGGAGCGUU	1498	chr1:173532542:173532617:-
m0075-3p	AUCACAUUGCCAGGGAUUUCCAA	1482	chr19:13947397:13947477:-
m0072-3p	ACAGUAGUCUGCACAUUGGUUAG	959	chr19:10928094:10928177:-
m0073-3p	UGGCUCAGUUCAGCAGGAACAGGG	946	chr19:13947091:13947177:-
m0143-3p	AGCAGCAUUGUACAGGGCUAUGA	934	chr5:167987899:167987981:-

### Differentially expressed profiles of miRNAs in penile cancers

When comparing the distinct levels of miRNA abundance between normal and cancerous tissues, two quantities are frequent of great importance: one is the p-value corresponding to the t-test responses to the issue whether the statistically significant difference of expression levels between the paired groups can be achieved. The other one is the fold-change (FC) value, which could illustrate what is the magnitude of the miRNA abundance difference between the paired specimens. Notably, even very large FC values may have insignificant p-values according to the t-test caused by considerable variabilities. Similarly, relatively small magnitude of the difference may also mean a tiny p-value due to the highly consistent technical replicates within each samples. Thus, a useful visualization method called “volcano plot”, which can simultaneously exhibit fold-change and p-values on the X-axis and Y-axis separately, has been introduced to conduct our analysis. Generally, points located in the upper-left or upper-right regions of the plot and indicated with the green color should draw more attention, as they had both small p-values (p<0.05) and high FC values (FC≥2). In this comparative study, the overview of the volcano plot generated by miRNAs profiles in penile cancer and matched normal tissues were shown (S4 Fig). As a result, significantly differential expression levels of 98 miRNAs were found between PeCa and adjacent normal penile specimens after adjustment for multiple testing. By applying additional filtering with a stringent cut-off of 50 reads count in both samples, we identified 56 miRNAs consisted of 30 (53.6%) miRNAs that were downregulated (Table 3) and 26 (46.4%) that were upregulated (Table 4) in the patients and could discriminate penile carcinoma from matched normal tissues.

**Table 3:** A collection of downregulated miRNAs detected by deep sequencing in cancerous penile tissues compared with the matched adjacent normal penile tissues.

miRNA name	Adjacent normal penile tissues		Cancerous penile tissues		Fold change	P value	FDR
	Absolute count	Relative count (rpm^a^)	Absolute count	Relative count (rpm^a^)			
hsa-miR-508-3p	133	9.6399	0	0	∞	7.23E-31	1.45E-30
hsa-miR-513c-5p	116	8.4077	0	0	∞	4.45E-27	7.85E-27
hsa-miR-509-3-5p	1494	108.2853	2	0.1423	760.78	0	0
hsa-miR-509-3p	535	38.7769	1	0.0712	544.87	2.02E-119	6.07E-119
hsa-miR-365b-3p	1489	107.9229	3	0.2134	505.49	0	0
hsa-miR-211-5p	887	64.2899	3	0.2135	301.12	4.47E-196	1.49E-195
hsa-miR-509-5p	85	6.16081	1	0.0712	86.57	1.64E-19	2.58E-19
hsa-miR-1247-5p	418	30.2967	94	6.6897	4.53	1.57E-47	4.28E-47
hsa-miR-204-3p	56	4.0589	15	1.0675	3.80	1.40E-06	1.62E-06
hsa-miR-205-5p	22994	1666.6076	6431	457.6783	3.64	0	0
hsa-miR-204-5p	617	44.7202	214	15.2299	2.94	8.01E-46	1.85E-45
hsa-miR-874-3p	145	10.5096	52	3.7007	2.84	2.34E-11	3.19E-11
hsa-miR-3065-5p	181	13.1189	66	4.6971	2.79	1.39E-13	2.08E-13
hsa-miR-320a	54859	3976.1862	20787	1479.3591	2.69	0	0
hsa-miR-887-3p	145	10.5096	55	3.9142	2.68	1.34E-10	1.75E-10
hsa-miR-505-3p	50	3.6240	20	1.4234	2.55	3.96E-04	3.96E-04
hsa-miR-328-3p	63	4.5662	26	1.8504	2.47	9.53E-05	1.02E-04
hsa-miR-145-5p	27272	1976.6775	11349	807.6801	2.45	0	0
hsa-miR-3184-3p	6563	475.6869	2782	197.9880	2.40	0	0
hsa-miR-495-3p	854	61.8980	364	25.9045	2.39	1.39E-46	3.49E-46
hsa-miR-23b-3p	21745	1576.0799	9665	687.8340	2.29	0	0
hsa-miR-30b-5p	626	45.3725	285	20.2827	2.24	8.18E-31	1.53E-30
hsa-miR-598-3p	431	31.2389	197	14.0200	2.23	1.62E-21	2.69E-21
hsa-miR-505-5p	65	4.7112	30	2.1350	2.21	3.47E-04	3.59E-04
hsa-miR-339-5p	103	7.4655	48	3.4160	2.19	6.58E-06	7.31E-06
hsa-miR-423-5p	28638	2075.6853	13353	950.2998	2.18	0	0
hsa-miR-141-3p	205	14.8584	100	7.1168	2.09	9.66E-10	1.21E-09
hsa-miR-320b	952	69.0010	476	33.8757	2.04	3.66E-38	7.84E-38
hsa-miR-200b-5p	263	19.0623	132	9.3941	2.03	1.78E-11	2.54E-11
hsa-miR-95-3p	173	12.5391	87	6.1916	2.03	5.99E-08	7.19E-08

^a^rpm: reads count per million reads count

**Table 4:** A collection of upregulated miRNAs detected by deep sequencing in cancerous penile tissues compared with the matched adjacent normal penile tissues.

miRNA name	Adjacent normal penile tissues		Cancerous penile tissues		Fold change	P value	FDR
	Absolute count	Relative count (rpm^a^)	Absolute count	Relative count (rpm^a^)			
hsa-miR-107	2180	158.0066	31730	2258.1452	14.29	0	0
hsa-miR-7-5p	22	1.5946	178	12.6678	7.94	2.44E-27	6.35E-27
hsa-miR-1277-3p	10	0.7248	58	4.1277	5.69	1.86E-08	3.02E-08
hsa-miR-136-5p	16	1.1597	70	4.9817	4.30	1.80E-08	3.11E-08
hsa-miR-223-3p	1110	80.4529	4153	295.5587	3.67	0	0
hsa-miR-3615	15	1.0872	55	3.9142	3.60	4.54E-06	6.21E-06
hsa-miR-455-5p	38	2.7542	125	8.8959	3.23	3.60E-11	7.20E-11
hsa-miR-323a-3p	26	1.8845	78	5.5511	2.95	9.20E-07	1.33E-06
hsa-miR-423-3p	189	13.6987	553	39.3556	2.87	4.40E-39	1.43E-38
hsa-miR-424-3p	259	18.7723	747	53.1621	2.83	2.74E-51	1.19E-50
hsa-miR-144-3p	62	4.4938	178	12.6678	2.82	3.29E-13	7.13E-13
hsa-miR-187-3p	19	1.3771	52	3.7007	2.69	1.99E-04	2.24E-04
hsa-miR-1278	19	1.3771	51	3.6295	2.64	2.85E-04	3.08E-04
hsa-miR-421	24	1.7395	64	4.5547	2.62	4.66E-05	5.77E-05
hsa-miR-944	352	25.5130	930	66.1858	2.59	3.71E-56	1.93E-55
hsa-miR-183-5p	22	1.5946	58	4.1277	2.59	1.27E-04	1.50E-04
hsa-miR-584-5p	39	2.8267	102	7.2591	2.57	3.17E-07	4.85E-07
hsa-miR-199a-5p	231	16.7429	600	42.7005	2.55	7.18E-36	2.07E-35
hsa-miR-1260b	187	13.5538	468	33.3064	2.46	9.41E-27	2.23E-26
hsa-miR-340-5p	805	58.3465	1952	138.9190	2.38	4.09E-101	3.55E-100
hsa-miR-424-5p	715	51.8233	1691	120.3443	2.32	4.24E-84	2.76E-83
hsa-miR-335-5p	68	4.9286	159	11.3156	2.30	5.34E-09	9.92E-09
hsa-miR-493-5p	36	2.6093	83	5.9069	2.26	3.83E-05	4.98E-05
hsa-miR-589-5p	23	1.6670	53	3.7719	2.26	1.17E-03	1.17E-03
hsa-miR-450a-5p	24	1.7395	55	3.9142	2.25	9.87E-04	1.03E-03
hsa-miR-3120-5p	427	30.9490	923	65.6876	2.12	2.01E-39	7.47E-39

^a^rpm: reads count per million reads count

Among which, the most abundantly downregulated miRNAs in cancerous penile tissues were miR-320a, miR-205-5p, miR-145-5p, miR-423-5p and miR-23b-3p. Of the upregulated miRNAs, miR-107, miR-223-3p, miR-340-5p, miR-424-5p and miR-944 were the top 5 most abundantly expressed miRNAs in the penile cancer tissues, among which, miR-107 had an expression level in PeCa exceeding 30,000 reads count. Meanwhile, miR-107 was also the most significantly upregulated miRNA as the log_2_(Cancer/Normal Ratio) was 3.83708. Correspondingly, miR-508-3p was the most significantly downregulated miRNA as the expression of miR-508-3p was even not detected in PeCa.

### Altered miRNA expression in cancerous penile tissues validated by qRT-PCR

To validate the altered miRNA expression in PeCa profiled by smRNA-seq, qRT-PCR was performed on the penile cancer tissues and matched histologically normal tissues. Firstly, to exclude the possible individual differences between the samples subjected to the sequencing, the 10 paired penile specimens from the patients were also mixed as a pool for the validated assay. Subsequently, we randomly picked 5 upregulated and 5 downregulated miRNAs among the differentially expressed miRNAs to conduct the qRT-PCR analysis and the expression levels were presented using the normalized fold change of cancerous tissues versus normal tissues. The results revealed that the expression levels and altered expression tendency of the 10 deregulated miRNAs were quite consistent with the results obtained from NGS technology. From both techniques, miR-509-3p showed the largest fold-change of downregulated expression levels in cancerous penile tissues, and miR-107 possessed the largest fold-change of upregulated expression levels in cancerous penile tissues, which together indicated that the expression levels of miRNAs detected by smRNA-seq sequencing were reliable (S5 Fig). Moreover, to validate the altered miRNA expression pattern revealed by the NGS data in individual patients, we picked 4 deregulated miRNAs to conduct the qRT-PCR analysis and the expression levels were presented using the normalized fold change of cancerous tissues versus normal tissues in each patient, respectively. According to the result, we found that although the population-based effects and inherent differences inevitably emerged as the previous report [33], the tendency of the expression patterns was consistent with the NGS findings, as miR-107 and miR-223-3p showed the upregulated expression levels in cancerous penile tissues, while miR-1247-5p and miR-509-3p showed the downregulated expression levels in cancerous penile tissues, which again verified the smRNA-seq sequencing data (S6 Fig).

### GO enrichment analysis of genes differentially expressed in cancerous and normal penile tissues

The essential biological functions of the putative target genes were classified via the Gene Ontology system. Since individual genes were related to distinct GO terms, gene sets which showed the similar deregulated expression patterns in multiple cancers were thought to be functionally crucial to the tumorigenic processes [30]. In order to better understand the biological behaviors of deregulated known miRNAs, potential target genes and biological functions targeted by these miRNAs were predicted by using MicroCosm, TargetSpy and miRanda softwares [26–29] (S1 File). After adjusting the number of total genes count annotated by a specific GO term≥10, enriched ratio≥2.0 and FDR p-value<0.05 to obtain the most convincing result for enrichment analysis, the top 10 most enriched GO terms including biological processes, cellular components and molecular functions were presented in Table 5. Among which, the putative target genes of deregulated miRNAs between the cancerous and normal penile specimens appeared to be mostly associated with the enriched cellular components consisted of cytoskeletons such as adherens junction (which junction anchors transmembrane proteins and facilitates cell cytoskeletons attachment), stress fiber (which consists of short actin filaments), cell-cell junction (which communicating junction commonly connects adjacent cells). Meanwhile, the most enriched molecular function among the top 10 GO terms was beta-catenin binding (which functions as the interactive behaviour with beta-catenin in a selective and non-covalent manner). Besides, these putative target genes were tightly related to a wide range of biological processes including growth regulation (which extensively modulates the organism's development), cell shape regulation (which functions as the cell surface configuration commander), axonogenesis (which process generally modulates the axon's morphogenesis and shape determination), cyclin-dependent protein kinase activity regulation (which procedure regulates serine/threonine kinase activity comprehensively), peptidyl-serine phosphorylation (which process mediates the conversion of peptidyl-serine to peptidyl-O-phospho-L-serine) and positive angiogenesis regulation (which procedure facilitates the formation of blood vessels).

**Table 5:** Top 10 most enriched GO terms for predicted targets of differentially expressed miRNAs between cancerous and adjacent normal penile tissues.

GO number	GO process	Targeted genes	Enrichment ratio	P value	FDR
GO:0005912	Adherens junction	CDH2 EPB41L5 KIFC3	2.99	0.0029	0.0288
		SHROOM3 SYNM TBCD			
		TJP1 TMEM204 VCL			
		WNK3 WTIP			
GO:0001725	Stress fiber	FHOD1 MST1R MYH10	2.85	0.0058	0.0195
		MYH11 MYH9 MYL12A			
		SEPT9 TPM1 VCL ZYX			
GO:0000079	Regulation of cyclin-dependent protein kinase activity	CCND3 CCNE1 CCNY	2.32	0.0133	0.0265
		CDC25A CDC6 CDK5R1			
		CDKN2A CDKN2D			
		FAM58A GTPBP4			
		SERTAD1 SFN			
GO:0018105	Peptidyl-serine phosphorylation	CDK5R1 MAP3K10 PINK1	2.27	0.0274	0.0343
		PLK1 PRKD2 RPS6KA2			
		SBK1 SMTNL1 STK4			
		TTBK2			
GO:0008360	Regulation of cell shape	ARHGEF18 CDC42EP1	2.22	0.0123	0.0306
		CDC42EP2 CDC42EP4			
		CYFIP1 ICAM1 MYH10			
		MYH9 MYL12A PALM			
		RHOU SHROOM3 SPTA1			
GO:0008013	Beta-catenin binding	APC2 CARM1 CDH2 DVL3	2.17	0.0319	0.0354
		GRIN2B PROP1 PTPRU			
		SOX17 TCF7L1 VCL			
GO:0040008	Regulation of growth	ARMC10 BRD8 BRMS1L	2.16	0.0245	0.0335
		C20orf20 GAP43 NDNL2			
		NEDD9 SOCS3 SOCS4			
		SOCS6 SOCS7			
GO:0007409	Axonogenesis	AFG3L2 ALS2 BAI1	2.13	0.0052	0.0262
		BAIAP2 DCC DVL1 FZD1			
		IGF1R LPPR4 MYH10			
		NKX2-8 NOTCH1 NTN1			
		SLIT1 SLITRK4 SLITRK6			
		SNAP25 VAX2			
GO:0045766	Positive regulation of angiogenesis	ANGPTL3 BTG1 C6 F3	2.03	0.0417	0.0417
		GDF2 IL1A IL1B MMP9			
		PLCG1 PRKD2			
GO:0005911	Cell-cell junction	AQP3 BAI1 CADM3 CDH2	2.03	0.0177	0.0295
		CNKSR1 COL17A1 DSG2			
		FNDC1 LDB1 PTPRU			
		RPGRIP1L STX2 SV2A			
		TJP1 TLN2 VCL			

### KEGG pathway analysis of genes differentially expressed in cancerous and normal penile tissues

After GO analysis, the putative target genes of these miRNAs differentially expressed in cancerous and normal penile specimens were introduced to KEGG to conduct a pathway enrichment analysis, which eventually showed 22 target genes that were annotated for differentially expressed miRNAs, and 5 KEGG pathways were enriched. Based on the statistically significant definition with FDR p-values<0.05, the annotated most enriched pathways were found to be tightly linked to “dorso-ventral axis formation (GRK-EGFR)” (which modulates the key effectors in axis formation such as Rho and Notch) and “colorectal cancer” (which is involved in the carcinogenic signaling transductions of genomic alternations resulted from chromosomal and microsatellite instabilities) (Table 6).

**Table 6:** KEGG pathway analysis for predicted target genes of differentially expressed miRNAs between adjacent normal and cancerous penile tissues.

Pathway name	Targeted genes	Enrichment ratio	P value	FDR
hsa04320: Dorso-ventral axis formation (GRK-EGFR)	MAP2K1 MAPK3	3.4342	0.0383	0.0479*
	NOTCH1 SOS1			
hsa05020: Prion diseases	IL1A IL1B LAMC1	3.4342	0.0120	0.06
	MAP2K1 MAPK3			
	NOTCH1			
hsa05217: Basal cell carcinoma	APC2 DVL1 DVL3	2.3967	0.0353	0.0588
	FZD1 TCF7L1			
	WNT11 WNT8B			
hsa05210: Colorectal cancer	APC2 DCC JUN	2.3264	0.0393	0.0393*
	MAP2K1 MAPK3			
	SMAD4 TCF7L1			
hsa04520: Adherens junction	BAIAP2 IGF1R MAPK3	2.2581	0.0329	0.0823
	SMAD4 SNAI1 TCF7L1			
	TJP1 VCL			

* The key KEGG pathways were enriched when FDR p-values<0.05 and enrichment ratio≥2.0 simultaneously.

All together, these enriched pathways participated in cell cycle, cell survival, proliferation, growth, differentiation, genomic instability and also apoptotic activity. Especially, we have summarized the specific signaling pathways participated in the comprehensive modulation of the putative target genes of the differentially expressed miRNAs between the cancerous and adjacent normal penile tissues, as follows: TGF-beta signaling pathway, Wnt signaling pathway, MAPK signaling pathway, p53 signaling pathway, PI3K-Akt signaling pathway and Notch signaling pathway (Detailed regulatory networks were available at KEGG pathway database on http://www.genome.jp/kegg/pathway.html [31]).

## Discussion

Penile cancer is associated with a number of potential risk factors including HPV infection, phimosis with chronic inflammation, poor hygiene and smoking, which disease predominantly affects aged males between 50 and 70 years old in most developing countries by high morbidity and mortality [34–35]. Although some oncogenes, chromosomal aberrations, epigenetic alterations, regulatory pathways and several core machineries involved in the penile cancer carcinogenesis, progression and invasion have been identified over the past decades [36–37], fine-tuning of the definite molecular concepts of penile cancer and especially the exact mechanisms involved in the development and progression of this highly mutilating disease remain largely pending. In-depth researches to further elucidate the detailed molecular events underlying the initiation and progression of penile cancer and its regulatory signaling pathways are eagerly necessary to direct prevention, early detection, targeted therapy and prognosis prediction. Notably, an increasing number of studies indicated that tumorigenesis, progression and invasion were regulated transcriptionally and post-transcriptionally in a strict and also cooperative manner by small non-protein-coding RNAs [38–39]. Among the variety kinds of smRNAs, microRNA (miRNA) molecules are a class of evolutionally conserved and endogenously expressed RNAs, which have been emerging as critical regulators that function in gene expression at posttranscriptional level [40–41]. Accumulating evidence has indicated that particular miRNAs either act as tumor oncogenes or suppressors, whose loss or overexpression aberrantly, could not only differentiate cancers arising from distinct physiological sites but also possess considerable prognostic significances [42–43]. Accordingly, as malignant tumors have shown additional traits beyond the acquisition of enhanced growth potency, decreased cell death, sustained angiogenesis as well as the invasion and metastasis ability, it is convincing that miRNAs could also act as master regulators of cancer therapy [44]. Thus, to generate the differentially expressed profiles of smRNAs in human penile cancer tissues and their matched adjacent normal tissues is prerequisite for thorough understanding of the definite regulatory roles of non-coding RNAs in penile cancer initiation, progression and invasion processes.

Recently, the newly developed next-generation sequencing (NGS) technology has been widely used for miRNA quantitative studies, by overriding the limitations in test specificity and inability to detect novel or low-abundance miRNAs such as rno-miR-509-5p/3p and rno-miR-1306-5p which can not be detected by the traditional quantitative assay [45]. Based on NGS, bioinformatic analyses of distinct miRNAs expression signatures and putative miRNAs binding sites have indicated several novel potential gene targets and signaling pathways of deregulated miRNAs in various cancers such as colorectal cancer [46] and breast cancer [47]. Particularly, for the genitourinary cancers, the comprehensively comparative miRNA expression profiles of prostate cancer [14, 48], bladder cancer [16], renal cancer [33], testicular germ cell tumors [49] and adrenocortical tumors [50] have been well-documented. Whereas, no any systematic studies have been conducted on miRNAs profiling in human penile cancer by using deep sequencing or even microarray.

In this present study, via utilizing state-of-the-art next-generation sequencing technology on smRNA libraries prepared from ten human penile cancer tissues and their matched adjacent tissues confirmed by the histopathologic diagnosis and then sequenced by employing the same protocols on the identical platform to guarantee the negligible inter-laboratory or inter-platform technical variations [18, 49], we have quantified 14,051,355 and 13,796,889 total sequencing reads aligned to the human genome sequence dataset, which represented 787,447 and 704,514 unique tags for the cancerous and adjacent normal tissues. Among these unique tags, 806 and 751 genome-widely expressed known miRNAs accompanied with 211 and 189 novel miRNAs were identified in PeCa and normal penis, respectively. Therein, the majority of highly expressed miRNAs were let-7-5p miRNA family members, which expression pattern was extremely similar as the miRNA profile in human testis [17, 51]. The possible explanation of the consistence was that mature form of let-7 family were highly conserved as their critical roles in developmental regulation of human reproductive organs including penis and testis were decided [52].

Furthermore, based on the "volcano plot" method which defined small p-values (p<0.05) and high fold change values (FC≥2) simultaneously and FDR adjustment, we have promoted this comparative study by focusing on exploring the miRNAs which showed the significantly aberrant expression pattern in penile cancer compared with matched histologically normal tissues, as differentially-expressed miRNAs were widely considered to be attractively novel prognostic biomarkers candidates and available targets for the early theragnosis of penile cancer. As a result, quantitatively differential expression analysis allowed the identification of a 56-miRNA signatures distinguishing penile cancer from normal penis. To make these signatures more concrete, the most commonly-used assay qRT-PCR was applied to validate the results obtained from NGS [14–18, 53]. Consequently, both the randomly-selected downregulated and upregulated miRNAs showed the almost unanimous altered expression pattern with the NGS data, indicating the miRNA profiles detected by the high-throughput sequencing technology were reliable.

Among the deregulated miRNAs, the most abundantly downregulated and upregulated miRNAs were miR-320a and miR-107, respectively. In consideration of the transformation of normal cells into malignant cancer cells may be governed by several common rules and undergo the identical biological processes [54], we have compared the deregulated miRNA expression patterns with other cancers with great interest. To begin with the most downregulated miRNA in penile cancer miRNA-320a, we found it often played as a tumor-suppressive miRNA in bladder cancer [55] and colorectal cancer [56] by targeting different oncogenes. Referring to the most abundantly and also significantly upregulated miRNA in penile cancer miR-107, it was consistently revealed the tumorigenic and metastatic potency to human colorectal cancer [57], pancreatic cancer [58] and gastric cancer [59]. Meanwhile, miR-508-3p, the most significantly downregulated miRNA in penile cancer, has been revealed a lower expression level in renal cancer tissues, in the same study, another downregulated miRNA named miR-509-3p which can suppress the proliferation of renal cancer cells was also found to be downregulated in penile cancer, together indicating its tumor suppressive role in cancer development [60].

To lend more concrete proofs to identify the potential modulatory roles of the miRNAs aberrantly expressed in penile cancer, we compared their expression patterns from NGS with previous reports in additional kinds of cancers. As expected, most majority of the miRNAs, either upregulated in the cancer tissues, such as miR-223-3p [61], miR-421 [62], miR-424-5p [63], miR-1260b [64] etc, or downregulated in the penile cancer, such as miR-205-5p [65], miR-211-5p [65–66], miR-365-3p [67] and miR-1247 [68] etc, were entitled the similar roles in carcinogenesis of bladder cancer, retinoblastoma, breast cancer, nasopharyngeal cancer, pancreatic cancer and several other cancer types [61–68]. Moreover, as miRNAs are highly tissue specific and can act as both tumor promoters or suppressors [69], we have then illuminated the definite roles of some “Janus miRNAs” in penile carcinogenesis regulation. For instance, expression of miR-23b-3p in cancer is somewhat controversial because it was either upregulated and oncogenic in renal cancer [70] or downregulated and execute suppressive effect in prostate cancer [71] and bladder cancer [72]. Similarly, recent studies have shown that miR-199a-5p played opposite roles in cancer initiation and development of various cancer types [73]. Our NGS data have revealed that miR-23b-3p was downregulated and miR-199a-5p was upregulated in penile cancer tissues, indicating their tumor-suppressive and oncogenic effect, respectively. More importantly, we have determined the regulatory function of several fresh miRNAs, including miR-887-3p, miR-1277-3p, miR-3120-5p etc, whose roles in carcinogenesis or cancer progression were largely unknown before.

miRNA databases enabled us to obtain the various currently validated targets of the differentially expressed miRNAs identified by our NGS data. Predicted target analyses suggested participation of the deregulated miRNAs in a number of biological processes involved in cell growth, axonogenesis, cell shape, protein activity regulation and angiogenesis. Accordingly, these top enriched GO terms were closely related to transformation of normal cells to malignant lesions. For instance, CDKN2A and MMP-9 among the putative target genes were proven to play crucial roles in penile carcinogenesis [5], notably, these two genes were successfully enriched in the regulation of cell growth, cyclin-dependent protein kinase activity and angiogenesis, respectively. Besides, IL-1A and IL-1B, which have been identified as the key machineries in inflammation modulation to cause penile cancer [3–4], were also successfully enriched in the current GO terms. Meanwhile, a remarkable GO term named axonogenesis, which was not usually associated with carcinogenesis but always played vital roles in the generation and outgrowth of axons during neuron development, was surprisingly enriched. Intriguingly, axonogenesis has been a recently described phenomenon of paramount importance in prostate cancer by participating in the proliferation and hormonal regulation of prostate cancer [74]. Therefore, our exploratory result has listed the predicted target genes and hinted a similar role of axonogenesis involved in penile cancer progression. Simultaneously, we have addressed that the enriched cellular components and molecular functions mainly consisted of cytoskeletal structures such as cell junctions, stress fibers and relevant beta-catenin binding regulation, respectively. This was reasonable since cellular morphogenesis, which determined cell shape and polarity, cell-cell junctions as well as cell-matrix adhesions through their interactions with cadherins and integrins, has been well established to accompany progression and invasion after carcinogenesis [75].

According to the KEGG pathway analysis, the tightly cancer-associated pathway “colorectal cancer” was successfully enriched. From which, the containing TGF-β [76], Wnt [77], MAPK [78] PI3K-Akt [79], p53 [80] and Notch signaling pathways [81] were previously documented to be involved in cancer development, progression, invasion, metastasis and also promising therapeutic targets screening. Besides, another pathway was also enriched. Although it was not usually considered as the direct carcinogenesis-modulated networks, the core molecular machinery Rho employed in the pathway was also definitely related with cancer biology [82]. Thus, our results have provided additional in-depth insights into the system-level molecular mechanisms of the tumorigenesis of penile cancer.

In addition, we have identified a distinct subset of 23-28-nt length smRNAs named piRNAs, which also differentially expressed in human penile cancers versus their matched adjacent normal penile tissues. These piRNAs are suggested to bind PIWI proteins to mediate posttranslational control of transposable elements expression and modulatory a crucial role in epigenetic changes via interaction with crucial proteins such as MAEL [83]. piRNAs have previously identified as smRNAs expressed predominantly in the germline of mammals [17, 84]. However, there were mounting evidences revealed piRNAs pathway activities in various cancers including breast cancer, gastrointestinal tract cancer, endometrioma and ovarian cancer recently [85–86]. For instance, piR-651 expression was upregulated in gastric cancerous tissues compared with paired non-cancerous tissues and the piR-651 level was further associated with cancer TNM stage, indicating piRNAs may also act as potential biomarkers for various cancer diagnosis [86]. Thus, in the present study, we have summarized the top 10 most abundant piRNAs in cancerous and adjacent normal penile tissues (S4 Table). From which, we found 6 out of 10 top abundant piRNAs expressed in paired penile tissues, again indicated the tissues with identically histological origin possessed the similar expression profiles of the smRNAs (Table 1). Simultaneously, we have compared the differentially expressed piRNAs in the paired penile tissues via a stringent cut-off of 50 reads count in either sample under the visualization "volcano plot" method (S5 Table and S7 Fig). Future studies will help to elucidate the detailed biogenesis and regulatory functions of these piRNAs in penile cancer initiation and progression.

Another potential advantage of our sequencing effort was the capability to evaluate possible miRNA modification events. Several studies have described miRNAs were modified to a significant degree in adult mouse brain and human testis, which modifications may enhance the miRNA stability, reinforce the specific miRNA-mRNA interactions and also execute some regulatory behaviors [17, 87]. In our current study, we also found some miRNAs from human penile tissues with similar modification profiles, among which, 6 modifications were overlapped in the top ten “5’ end added” modifications in both of the paired specimens, indicating the same tissue origin not only possessed the similar expression profiles of the smRNAs, but also showed the highly similar modification pattern [18]. Intriguingly, some miRNAs such as miR-320a, which was differentially expressed between the cancerous and normal penile tissues (Table 3), possessed slightly different modification behaviors between the paired specimens as well (S6 and S7 Tables). The distinct modifications including adding or trimming in each 5’ or 3’ terminus of the deregulated miRNAs expressed in penile cancers and normal adjacent tissues might be related to the carcinogenesis, which findings also provided promising avenues for the design of innovative strategies in fighting against penile cancer.

## Conclusions

In summary, to the best of our knowledge, we have obtained the first data for the comprehensive characterizations of miRNAs in penile cancer by next-generation deep sequencing, which technology enabled to precisely quantify expression levels of deregulated miRNAs that may serve as attractively novel diagnostic markers in PeCa. Several differentially expressed miRNAs potentially target networks of genes and signaling pathways probably be involved in the malignant transformation of normal penis to PeCa. Besides, we also explored the novel miRNAs and deregulated piRNAs in cancerous and matched adjacent normal penile tissues. Our data provide an important platform for future investigations aimed to characterize the concrete roles of specific miRNAs and other regulatory smRNAs including piRNAs in penile cancer initiation, progression, invasion and also effective theragnosis. Notably, further studies to conduct the deep sequencing in separated rather than pooled clinical specimens may offer considerable benefits to such efforts.

## Supporting Information

S1 Fig Length distribution of clean reads from smRNA next-generation deep sequencing. The most majority of these clean reads were 22 nt in size, consistently followed by 23 nt and 21 nt in both cancerous and adjacent normal penile tissues.
(TIF)

S2 Fig Number of unique small RNA sequencing tags that locate on each chromosome. Chromosome 1 harbored most of the unique tags, followed by chromosome 2, 17, 12, 11 in adjacent normal penile tissues and the unique tags located on chromosome 1, 2, 11, 12, 17 of the cancerous penile tissues in decreasing order.
(TIF)

S3 Fig The folding secondary structures of novel miRNAs in adjacent normal and cancerous penile tissues. Precursors strictly matched the default parameters for novel miRNAs were identified and the potential stem-loop structures were shown.
(TIF)

S4 Fig The overview of the volcano plot generated by miRNAs profile in adjacent normal penile tissues and cancerous penile tissues. The vertical lines corresponded to 2-fold up and down-regulation, respectively, and the horizontal line represented a p-value of 0.05. The blue points in the plot represented the differentially expressed miRNAs with statistical significance while the red points in the plot represented the similarly expressed miRNAs without statistical significance.
(TIF)

S5 Fig Confirmation of differentially expressed miRNAs between cancerous and adjacent normal penile tissues obtained by NGS using qRT-PCR. Validation of the relative expression levels of ten deregulated miRNAs in cancerous and adjacent normal penile tissues (each five for downregulated and upregulated miRNAs, respectively). The relative expression levels of miRNAs between the matched pooled samples were shown with the expression fold change (cancer/normal) for comparing the NGS data with the qRT-PCR results.
(TIF)

S6 Fig Detection of representatively differentially expressed miRNAs in individual penile cancer patients. Validation of the relative expression levels of four deregulated miRNAs in cancerous and adjacent normal penile tissues of individual patients (each two for downregulated and upregulated miRNAs, respectively). The relative expression levels of miRNAs between the paired samples were shown with the expression fold change (cancer/normal).
(TIF)

S7 Fig The overview of the volcano plot generated by piRNAs profile in normal and cancerous penile tissues. The vertical lines corresponded to 2-fold up and down-regulation, respectively, and the horizontal line represented a p-value of 0.05. The blue points in the plot represented the differentially expressed piRNAs with statistical significance while the red points in the plot represented the similarly expressed piRNAs without statistical significance.
(TIF)

S1 File The putative target genes of deregulated miRNAs between cancerous and adjacent normal penile tissues. (XLS)

S1 Table Primers used for the quantification of representative deregulated miRNAs in adjacent normal and cancerous penile tissues. (XLS)

S2 Table The match results of clean reads in adjacent normal and cancerous penile tissues. (XLS)

S3 Table The repeats types based on the unique tags and total reads count. (XLS)

S4 Table The list of top 10 most abundant known piRNAs expressed in adjacent normal penile tissues and cancerous penile tissues. (XLS)

S5 Table Differentially expressed piRNAs in adjacent normal and cancerous penile tissues. (XLS)

S6 Table List of top 10 miRNAs with possible modification events in adjacent normal penile tissues. (XLS)

S7 Table List of top 10 miRNAs with possible modification events in cancerous penile tissues. (XLS)
