# Supplementary figures and images for: MicroRNA Expression Profile in Penile Cancer Revealed by Next-Generation Small RNA Sequencing
Source: PLoS One. 2015 Jul 9;10(7):e0131336. doi: 10.1371/journal.pone.0131336 (PMC4497725; doi:10.1371/journal.pone.0131336)

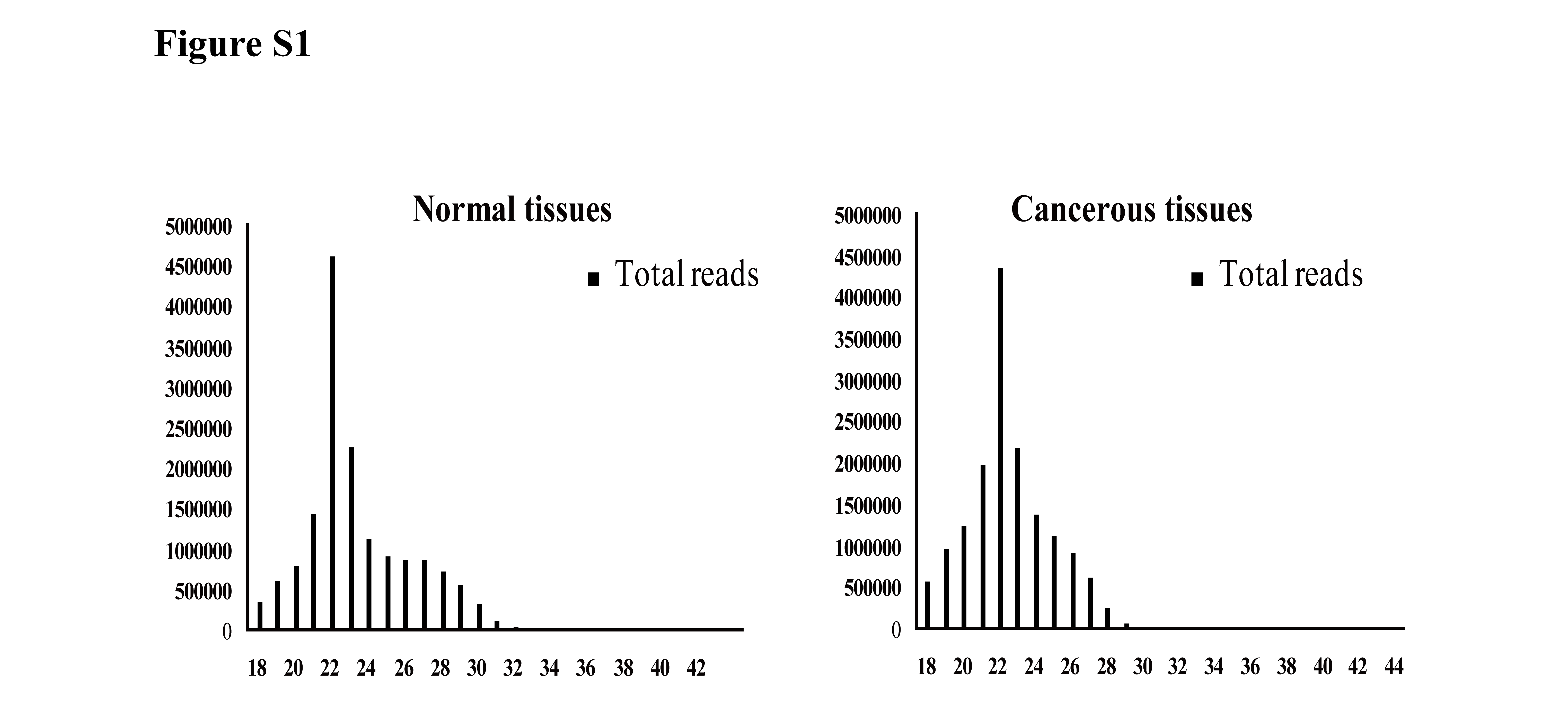

Supplement: S1 Fig — The most majority of these clean reads were 22 nt in size, consistently followed by 23 nt and 21 nt in both cancerous and adjacent normal penile tissues. (TIF) [file pone.0131336.s001.tif]

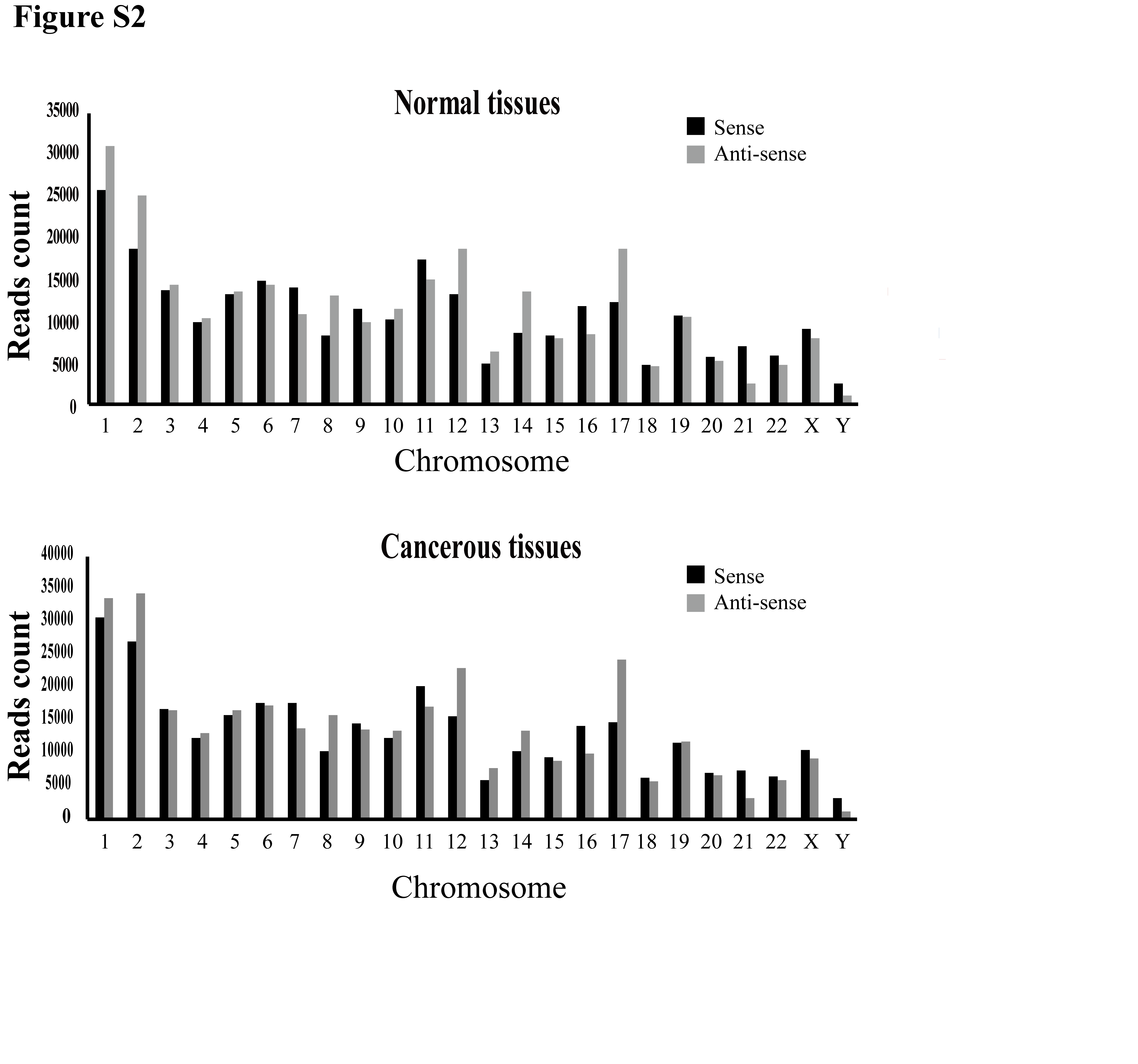

Supplement: S2 Fig — Chromosome 1 harbored most of the unique tags, followed by chromosome 2, 17, 12, 11 in adjacent normal penile tissues and the unique tags located on chromosome 1, 2, 11, 12, 17 of the cancerous penile tissues in decreasing order. (TIF) [file pone.0131336.s002.tif]

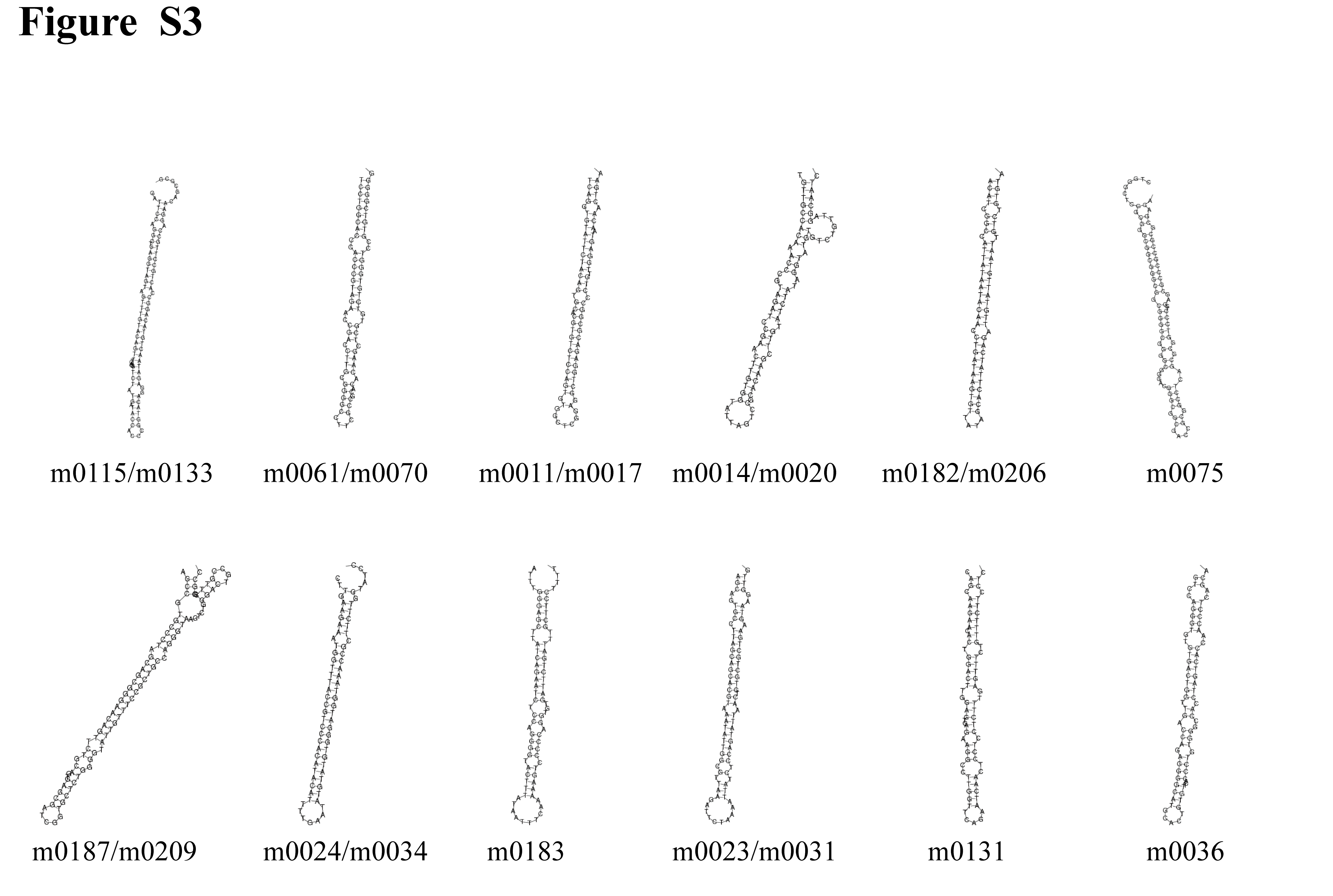

Supplement: S3 Fig — Precursors strictly matched the default parameters for novel miRNAs were identified and the potential stem-loop structures were shown. (TIF) [file pone.0131336.s003.tif]

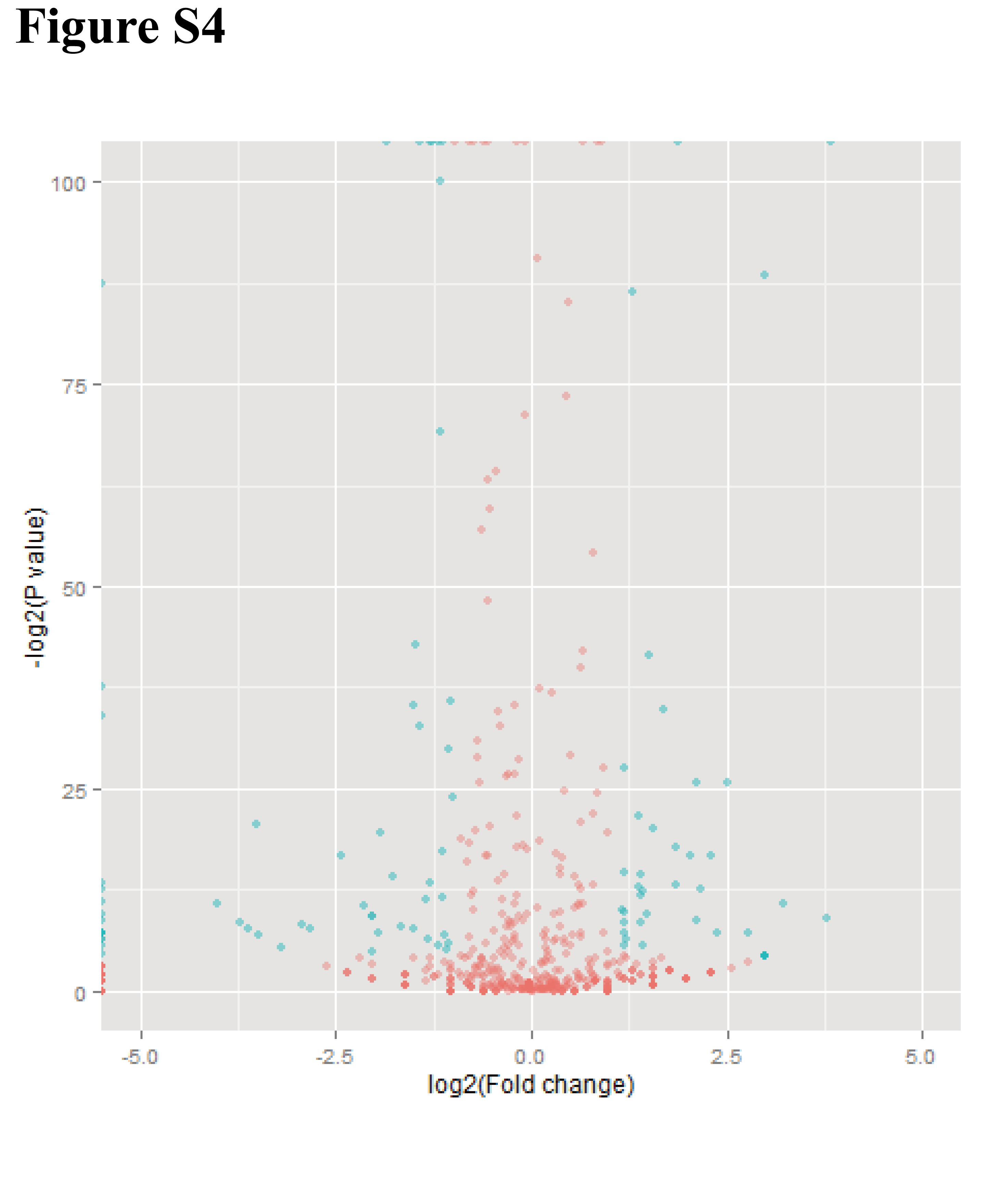

Supplement: S4 Fig — The vertical lines corresponded to 2-fold up and down-regulation, respectively, and the horizontal line represented a p-value of 0.05. The blue points in the plot represented the differentially expressed miRNAs with statistical significance while the red points in the plot represented the similarly expressed miRNAs without statistical significance. (TIF) [file pone.0131336.s004.tif]

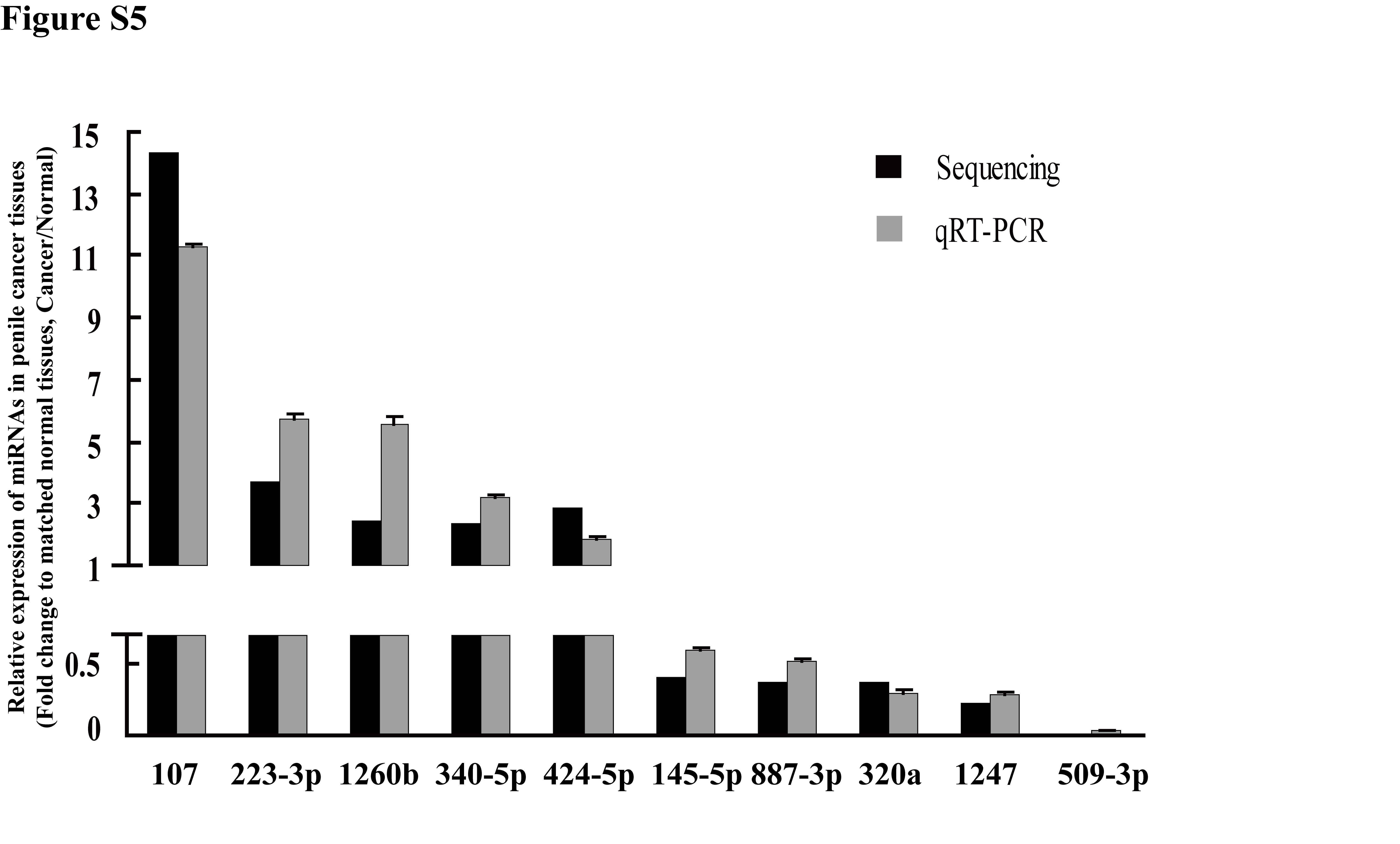

Supplement: S5 Fig — Validation of the relative expression levels of ten deregulated miRNAs in cancerous and adjacent normal penile tissues (each five for downregulated and upregulated miRNAs, respectively). The relative expression levels of miRNAs between the matched pooled samples were shown with the expression fold change (cancer/normal) for comparing the NGS data with the qRT-PCR results. (TIF) [file pone.0131336.s005.tif]

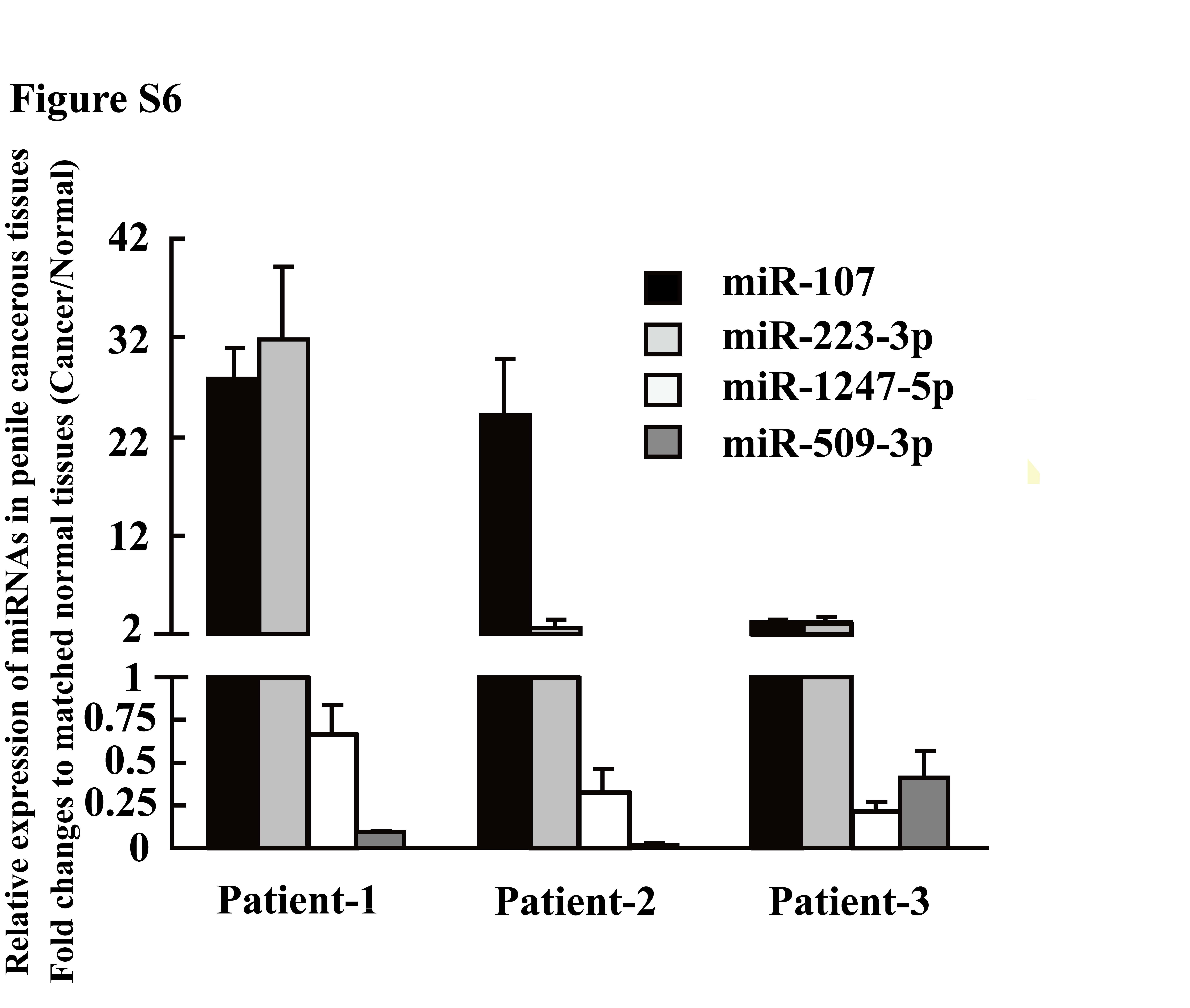

Supplement: S6 Fig — Validation of the relative expression levels of four deregulated miRNAs in cancerous and adjacent normal penile tissues of individual patients (each two for downregulated and upregulated miRNAs, respectively). The relative expression levels of miRNAs between the paired samples were shown with the expression fold change (cancer/normal). (TIF) [file pone.0131336.s006.tif]

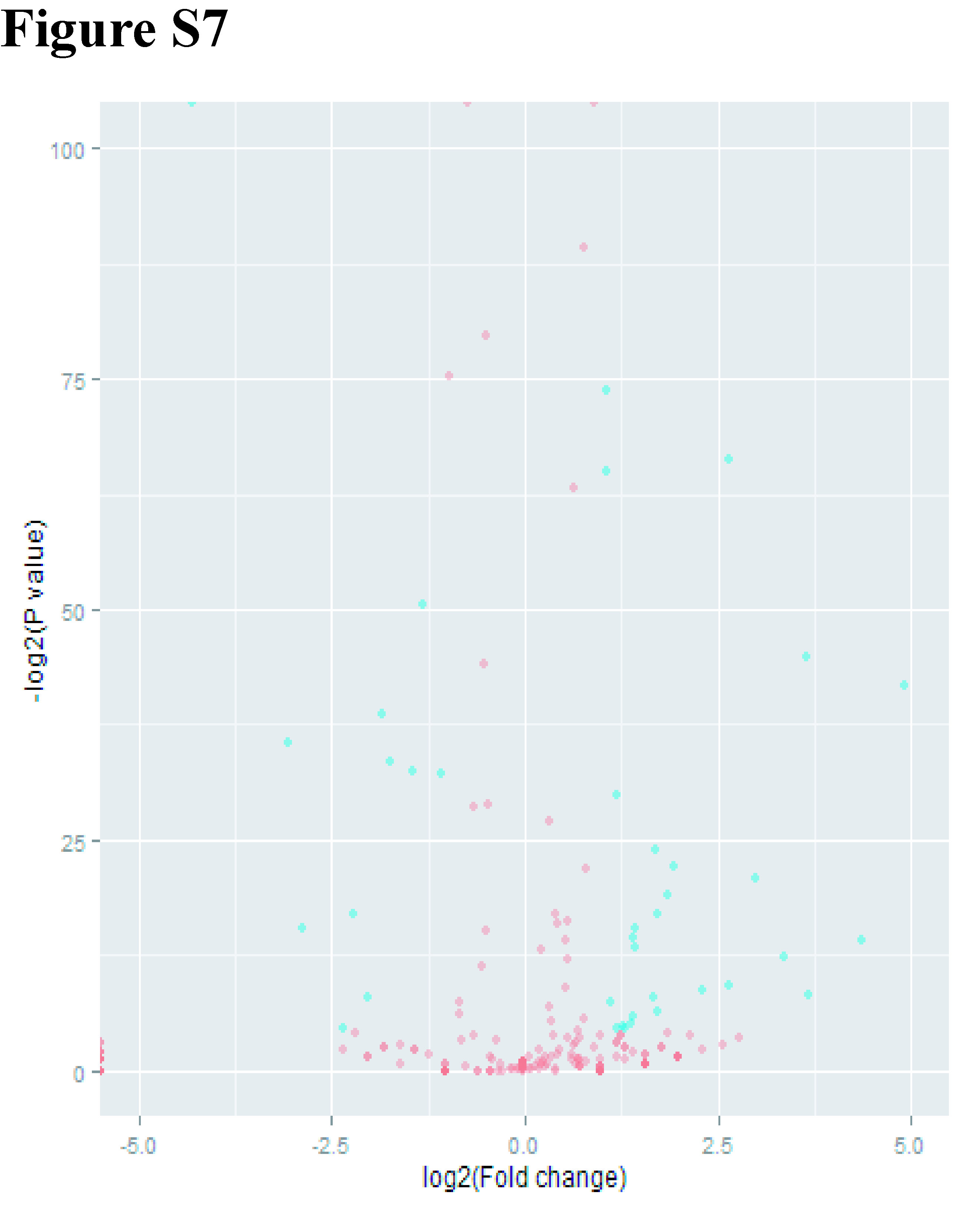

Supplement: S7 Fig — The vertical lines corresponded to 2-fold up and down-regulation, respectively, and the horizontal line represented a p-value of 0.05. The blue points in the plot represented the differentially expressed piRNAs with statistical significance while the red points in the plot represented the similarly expressed piRNAs without statistical significance. (TIF) [file pone.0131336.s007.tif]
